# Recurrent ischemic stroke in patients with atrial fibrillation ablation and prior stroke: A study based on etiological classification

**DOI:** 10.1002/joa3.12285

**Published:** 2019-12-03

**Authors:** Seiji Fukamizu, Rintaro Hojo, Takeshi Kitamura, Iwanari Kawamura, Satoshi Miyazawa, Jun Karashima, Shin Nakamura, Kosuke Takeda, Koichiro Yamaoka, Tomoyuki Arai, Kohei Kawajiri, Sho Tanabe, Yasuki Koyano, Daisuke Miyahara, Sayuri Tokioka, Marina Arai, Dai Inagaki, Tomonori Miyabe, Harumizu Sakurada, Masayasu Hiraoka

**Affiliations:** ^1^ Department of Cardiology Tokyo Metropolitan Hiroo Hospital Tokyo Japan; ^2^ Department of Cardiology Tokyo Metropolitan Health and Hospitals Corporation Ohkubo Hospital Tokyo Japan; ^3^ Tokyo Medical and Dental University Tokyo Japan

**Keywords:** atrial fibrillation, catheter ablation, cerebral infarction, pulmonary vein isolation, TOAST classification

## Abstract

**Background:**

Different subtypes of ischemic stroke may have different risk factors, clinical features, and prognoses. This study investigated the incidence and mode of stroke recurrence in patients with a history of stroke who underwent atrial fibrillation (AF) ablation.

**Methods:**

Of 825 patients who underwent AF ablation from 2006 to 2016, 77 patients (9.3%, median age 69 years) with a prior ischemic stroke were identified. Patients were classified as those with prior cardioembolic (CE) stroke (n = 55) and those with prior non‐CE stroke (n = 22). The incidence and pattern of stroke recurrence were investigated.

**Results:**

The incidence of asymptomatic AF (54.5% vs 22.7%; *P* = .011) and left atrial volume (135.8 mL vs 109.3 mL; *P* = .024) was greater in the CE group than in the non‐CE group. Anticoagulation treatment was discontinued at an average of 28.1 months following the initial ablation in 34 (44.2%) patients. None of the patients developed CE stroke during a median 4.1‐year follow‐up. In the non‐CE group, 2 patients experienced recurrent non‐CE stroke (lacunar infarction in 1 and atherosclerotic stroke in 1); however, AF was not observed at the onset of recurrent ischemic stroke.

**Conclusions:**

In patients with a history of stroke who underwent catheter ablation for AF, the incidence of recurrent stroke was 0.54/100 patient‐years. The previous stroke in these patients may not have been due to AF in some cases; therefore, a large‐scale prospective study is warranted to identify the appro priate antithrombotic therapy for the prevention of potentially recurrent stroke.

## Abstract 2

We investigated stroke recurrence in patients with a prior stroke who underwent atrial fibrillation ablation. Of 77 patients, none developed cardioembolic (CE) stroke during a median follow‐up period of 4.1 years. Two patients (0.54/100 patient‐years) experienced recurrent non‐CE stroke. Therefore, the optimal antithrombotic therapy should be discussed in future studies.

## 1 INTRODUCTION

Atrial fibrillation (AF) increases the risk of a cerebrovascular event (CVE) up to 5 times.^1^ Patients with AF and history of stroke are at higher risk of death, heart failure, and long‐term disability. Catheter ablation is the standard procedure for treating patients with AF, and recent studies have suggested that it may further reduce the risk of thromboembolism.^2^, ^3^, ^4^ For antithrombotic therapy after catheter ablation for AF, current guidelines recommend continued oral anticoagulation (OAC) therapy based on the CHA_2_DS_2_‐VASc score risk profile for all patients.^5^ However, in clinical practice, OAC therapy has been discontinued for many patients with a low‐risk profile for thromboembolism. Very few studies have described outcomes in high‐risk patients with apparently successful AF ablation following discontinuation of OAC therapy. Furthermore, these studies did not provide details of the type of stroke experienced (ie, whether past CVEs were cardiogenic embolisms). We hypothesized that different subtypes of ischemic stroke may present different risk factors, clinical features, and prognosis; therefore, the best post‐procedural antithrombotic management for AF ablation may differ in patients with prior cardioembolic (CE) stroke and prior non‐CE (ie, non‐AF related) stroke. This retrospective, observational study investigated the incidence and mode of ischemic stroke recurrence in patients with a history of ischemic stroke that have undergone AF ablation.

## 2 METHODS

### 2.1 Study population

**Figure 1:**
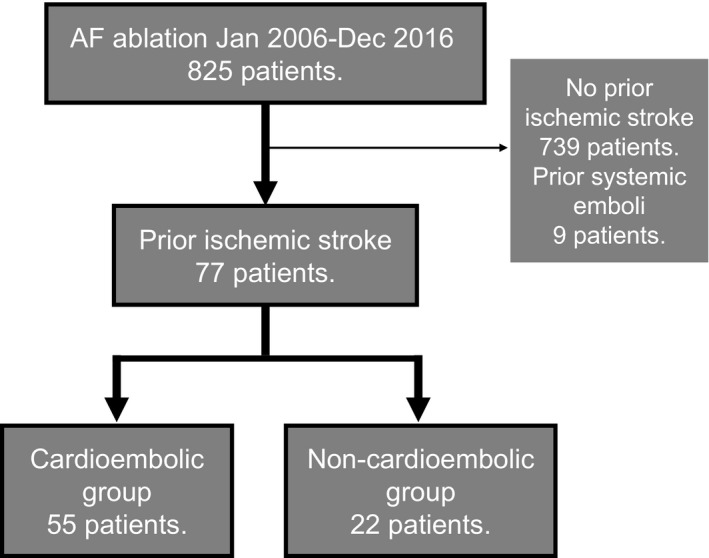
Flow diagram of the study patients. AF, atrial fibrillation

Of 825 consecutive patients who underwent AF ablation at the Tokyo Metropolitan Hiroo Hospital between 1 January 2006 and 31 December 2016, 77 patients (9.3%) with a prior cerebral infarction/transient ischemic attack (TIA) were identified (Figure 1). Retrospective information about the history of stroke (based on self‐report and patient records) and risk factors were extracted from clinical medical records. Nine patients with systemic emboli, considered a risk factor in the CHA_2_DS_2_‐VASc score, were excluded from this study (involvement of the peripheral or coronary artery in 6 and 3, respectively). All patients provided written informed consent before undergoing the procedures. Data collection was approved by the institutional review board of the hospital.

### 2.2 Classification of ischemic stroke

The causes of ischemic stroke were classified according to the Trial of Org 10172 in Acute Stroke Treatment (TOAST) classification.^6^ The findings specified in the classification of ischemic stroke were as follows: symptoms at onset, computed tomography (CT) or magnetic resonance imaging (MRI) findings in the acute phase, interpretation of images by radiologists or neurologists, and history of AF. If necessary, carotid artery ultrasonography, CT angiography, and magnetic resonance angiography were also performed. When multiple causes were considered, we classified them as more reasonable causes. Etiologies of ischemic stroke were classified as (a) large artery atherosclerosis; (b) CE; (c) lacunar; (d) stroke of other determined etiology; (e) stroke of undetermined etiology; and (f) TIA. Patients were divided into 2 groups: prior CE stroke (CE group) and non‐CE stroke (non‐CE group). The incidence and pattern of recurrence of stroke during the long‐term follow‐up in both groups were investigated.

### 2.3 Catheter ablation protocol

Our ablation protocol has been described previously.^7^ Briefly, transesophageal echocardiography was performed to exclude the formation of intra‐atrial thrombus the day before the procedure. All patients underwent circumferential isolation of the ipsilateral PVs at the antrum. A 3.5‐mm irrigated‐tip catheter (ThermoCool Navistar or ThermoCool Navistar STSF, Biosense Webster; or Cool Path, St. Jude Medical) and a three‐dimensional anatomical mapping system (CARTO, Biosense Webster; or Ensite, St. Jude Medical) were used for mapping and ablation. In cases using an ablation catheter with a contact force sensor, the contact force was controlled to achieve a >450 force‐time integral for each point. From December 2012, ablation with a cryoballoon was performed for patients with paroxysmal AF except for left common trunk and patients with chronic kidney disease or contrast agent allergy. For cryoablation, a 28 mm cryoballoon (Arctic Front Advance; Medtronic) was used with confirmation of PV occlusion with contrast injection. In general, each cryoablation was performed with a target ablation time of 180 seconds. If electrical isolation was not achieved despite up to 3 applications, touch‐up ablation was performed using an irrigated‐tip radiofrequency catheter.

The endpoint of PV isolation was the achievement of a bidirectional conduction block between the LA and PVs. After confirmation of the complete bidirectional block, continuous intravenous administration of isoproterenol (4 μg/min) was initiated, followed by a bolus injection of 40 mg of adenosine triphosphate to exclude reconduction or dormant conduction between the LA and the PVs. Catheter ablation was performed to eliminate the presence of reconnection and/or dormant conduction. If non‐PV foci were identified with drug infusions, catheter ablation was applied to the foci. When non‐PV foci were located in the superior vena cava (SVC), circumferential isolation of the SVC was performed. In patients with paroxysmal AF and persistent AF lasting for ≤1 year, circumferential PV antrum isolation was performed. In patients who had longstanding persistent AF (>1 year), PV isolation was performed, followed by mapping and ablation of complex fractionated atrial electrograms. Additionally, linear ablation of a roof and mitral isthmus line or left atrial posterior wall box isolation was performed at the physician's discretion.

### 2.4 Pre‐ and periprocedural anticoagulation

Oral anticoagulations at the time of ablation included warfarin in 53 patients and direct oral anticoagulants (DOACs) in 24 patients (Table 1). There was no significant difference in the type of OACs between the CE and non‐CE groups. Warfarin was not interrupted unless there was a marked prothrombin time‐international normalized ratio prolongation. All DOACs were suspended the morning of the procedure. During ablation, we administered 90 units/kg of heparin before the transseptal puncture, measured the activated clotting time every 10 minutes, and administered an additional dose of heparin to maintain activated clotting times between 350 and 400 seconds.

**Table 1:** Baseline clinical characteristics of patients with prior cardioembolic stroke and prior non‐cardioembolic stroke

	CE group n = 55	Non‐CE group n = 22	*P* value
Demographics			
Age (y)	67.3 ± 9.3	68.9 ± 9.8	.502
Age over 65 y old, n (%)	36 (66%)	18 (82%)	.181
Sex, female, n (%)	10 (18%)	7 (32%)	.229
Body mass index	23.5 ± 3.6	24.7 ± 3.7	.689
Former or current smoker, n (%)	34 (62%)	13 (59%)	1.000
Medical history			
Organic heart disease, n (%)	14 (26%)	3 (14%)	.366
Type of AF, non‐PAF, n (%)	13 (24%)	3 (14%)	.535
Congestive heart failure, n (%)	9 (17%)	2 (9%)	.497
Hypertension, n (%)	34 (62%)	18 (82%)	.090
Uncontrolled blood pressure over 160 mm Hg, n (%)	1 (2%)	4 (18%)	.022
Diabetes merits, n (%)	16 (29%)	5 (23%)	.571
Vascular disease, n (%)	10 (18%)	4 (18%)	1.000
Major bleeding history, n (%)	2 (4%)	1 (5%)	1.000
Asymptomatic AF, n (%)	30 (55%)	5 (23%)	.011
AF duration (mo; median [25th‐75th])	36 [14‐60]	28 [5‐70]	.604
CHADS_2_ score	3.3 ± 1.0	3.3 ± 1.0	.915
CHA_2_DS_2_‐VASc score	4.3 ± 1.3	4.6 ± 1.3	.260
CHA_2_DS_2_‐VASc score ≥ 4, n (%)	38 (69%)	18 (82%)	.396
HAS‐BLED score	1.9 ± 0.7	2.4 ± 0.7	.002
Medications			
Anticoagulants			.107
Warfarin, n (%)	41 (75%)	12 (55%)	
DOACs, n (%)	14 (26%)	10 (46%)	
Antiplatelet, n (%)	9 (16%)	8 (36%)	.072
Statin, n (%)	23 (42%)	12 (55%)	.326

Abbreviations: AF, atrial fibrillation; DOACs, direct oral anticoagulants; PAF, paroxysmal atrial fibrillation.

### 2.5 Repeat ablation procedure

Repeat catheter ablation was recommended in patients experiencing atrial tachyarrhythmia after the final procedure. Moreover, repeated sessions were recommended if the patient's symptoms strongly suggested recurrence of AF, even if AF could not be detected in the electrocardiogram. During repeat sessions, LA‐PV reconnection or a reproducibly induced non‐PV trigger was ablated at the reconnected PVs or non‐PV triggers. For patients who relapsed with persistent AF, a combination of roofline, mitral isthmus line, SVC isolation, and left atrial posterior wall box isolation was performed at the discretion of each physician. Re‐initiation of anti‐arrhythmic drugs (AADs) was considered when patients with AF recurrence did not undergo repeat procedure. Basically, OAC was reintroduced in the re‐session for recurrent AF in patients who have discontinued OAC therapy. After confirming the absence of AF recurrence after the last session, OAC was either terminated again or continued in some cases, and the decision was left to the discretion of the physician.

### 2.6 Follow‐up and clinical outcomes

Following the ablation procedure, all patients were discharged from the hospital with prescription of OACs. Recurrence of atrial tachyarrhythmia was evaluated based on the patient's symptoms, resting 12‐lead electrocardiogram findings at regular visits at the outpatient clinic, and 24‐hour Holter ambulatory monitoring results at 1, 3, 6 months, and every 6 months thereafter the final procedure. Recurrent atrial tachyarrhythmia was defined as a documented AF/flutter/tachycardia lasting >30 seconds without a blanking period. OAC discontinuation during follow‐up was permitted in selected patients, and restarting of OACs was allowed in cases with recurrent atrial tachyarrhythmia according to the physician's discretion. When patients were admitted for ischemic stroke at other institutions, medical history, and examination results were collected from the attending neurologists.

The following outcomes were compared: the primary outcome was the recurrence of ischemic stroke, and secondary outcomes were the occurrence of major bleeding events, the time to discontinuation of OACs from the time of initial catheter ablation, and recurrence of atrial tachyarrhythmias.

### 2.7 Statistical analysis

Continuous and categorical data are presented as mean ± SD or median (interquartile range [IQR]), as appropriate, and numbers and percentages (%), respectively. Continuous variable data were compared by the independent samples *t* test when data distribution was normal or by the Mann‐Whitney *U* test for non‐normal distributions. Categorical variables were analyzed using the *χ* ^2^ test; otherwise, the Fisher's exact test was used. We performed multivariable regression analyses to determine the association between stroke subtype (CE or non‐CE) and multiple variables of interest. The cumulative incidences of discontinuing OACs were analyzed using the Kaplan‐Meier method and log‐rank test. All analyses were conducted with SPSS version 18.0J (SPSS Inc). The level of significance was set at *P* < .05.

## 3 RESULTS

### 3.1 Baseline characteristics

In all, 77 patients (median age 69 [range 36‐85] years, 22% women, average CHADS_2_ score 3.3 ± 1.0 and CHA_2_DS_2_‐VASc score 4.4 ± 1.3, 61 paroxysmal, 9 persistent, 7 longstanding persistent) were followed for a median of 4.1 (IQR 2.6‐6.7, maximum 11.6) years. The OACs used initially were warfarin (n = 53), dabigatran (n = 6), rivaroxaban (n = 9), apixaban (n = 7), and edoxaban (n = 2).

### 3.2 Etiologies of ischemic stroke

The etiologies of prior stroke based on the TOAST classification were CE in 55 patients (71.4%), lacunar infarct in 11 (14.3%), large artery atherosclerosis in 4 (5.2%), stroke of other determined etiology in 1 (1.3%), stroke of undetermined etiology in 2 (2.6%), and TIA in 4 (5.2%). In total, 55 patients were assigned to the CE group and 22 to the non‐CE stroke group.

### 3.3 Risk factors and clinical features

Comparison of background risk factors and clinical parameters in the CE and non‐CE groups indicated no significant differences in age, sex, presence of organic heart disease, history of heart failure, diabetes, or vascular disease (Tables 1 and 2). History of hypertension tended to be higher in the non‐CE group (18/22 [81.8%] vs 34/55 [61.8%]; *P* = .090). No significant difference was found for the CHADS_2_ or CHA_2_DS_2‐_VASc scores in either group. The HAS‐BLED score was significantly higher in the non‐CE group than in the CE group (2.4 ± 0.7 vs 1.9 ± 0.7; *P* = .002). In the non‐CE group, there were significantly more patients with poor control of blood pressure. In addition, the proportion of elderly people aged over 65 years and the number of patients using antiplatelet drugs tended to be high in the non‐CE group. Moreover, no significant difference was found in the type of AF between the 2 groups. The proportions of asymptomatic AF (30/55 [54.5%] vs 5/22 [22.7%]; *P* = .011) and the left atrial volume (135.8 mL [IQR 106.1‐72.3] vs 109.3 mL [IQR 93.6‐137.3]; *P* = .024) were larger in the CE group than in the non‐CE group. Left atrial dimension, left ventricular ejection fraction, ratio of transmitral Doppler early filling velocity to tissue Doppler early diastolic mitral annular velocity (E/E′), and left atrial appendage flow velocity were comparable. No significant differences were found for laboratory data, including brain natriuretic peptide, in both groups. Multiple logistic‐regression analysis showed an independent association between asymptomatic AF and CE stroke (odds ratio, 4.35; 95% confidence interval, 1.267‐14.925; *P* = .020).

**Table 2:** Echocardiographic and laboratory data of patients with prior cardioembolic stroke and prior non‐cardioembolic stroke

	CE group n = 55	Non‐CE group n = 22	*P*‐value
Echo parameters			
LAD (mm)	40.9 ± 7.4	40.7 ± 9.1	.898
LVEF (%)	60.5 ± 10.2	65.5 ± 8.2	.047
E/e′ *	11.4 (9.0‐16.0)	11.1 (7.2‐13.1)	.353
LAV‐TTE (mL)^a^	65.5 (55.0‐89.0)	63.0 (38.1‐71.6)	.217
LAV‐CT (mL)^a^	135.8 (106.1‐172.3)	109.3 (93.6‐137.3)	.024
LAAFV (m/s)	49.7 ± 24.0	55.3 ± 18.0	.325
LAAFV ≤ 20 m/s, n (%)	3 (6%)	1 (5%)	1.000
SEC, n (%)	10 (18%)	3 (14%)	.747
PFO, n (%)	2 (4%)	1 (5%)	1.000
Laboratory parameters			
BNP (pg/mL)	183.1 ± 258.6	146.3 ± 221.8	.560
Creatinine (mg/dL)	0.9 ± 0.2	0.9 ± 0.3	.370
eGFR (mL/min/1.73 m^2^)	65.7 ± 19.9	68.2 ± 17.1	.609

Abbreviations: BNP, brain natriuretic peptide; GFR, estimated glomerular filtration rate; LAAFV, left atrial appendage flow velocity; LAD, left atrial dimension; LAV‐CT, left atrial volume measured by computed tomography; LAV‐TTE, left atrial volume measured by trans‐thoracic echocardiography; LVEF, left ventricular ejection fraction; PFO, patent foramen ovale; SEC, spontaneous echo contrast

a Mann‐Whitney *U* test, expressed by median [25th‐75th].

### 3.4 Ablation procedure and periprocedural complications

Overall, 144 ablation procedures in 77 patients were performed including a 2nd session in 35, a 3rd session in 11, a 4th session in 2, and a 5th session in 1 patient. In the initial session, radiofrequency catheter ablation was performed in 70 patients and cryoballoon ablation was performed in 7 patients. In all, 49 patients required a repeat procedure. Of these, 44 patients (89.7%) with reconnection of at least 1 PV underwent reisolation of reconnected PVs followed by additional ablation as needed. No significant complications were found except for 1 cardiac tamponade and 2 arteriovenous fistulas in the initial ablation procedure and 1 cardiac tamponade in the repeat procedure. None of the patients exhibited periprocedural thromboembolic events in any of the procedures.

### 3.5 Follow‐up outcomes

Despite multiple ablation procedures (1.8 per patient), the rhythm control strategy to maintain sinus rhythm was switched to rate control (followed as permanent AF) in 2 patients (2.6%). The remaining 75 patients (of which 21 patients were taking AAD for recurrent atrial tachyarrhythmia or concomitant ventricular arrhythmias) had no atrial tachyarrhythmia at the final observation. During a median follow‐up period of 30.5 (IQR 13.2‐57.5) months, sporadic atrial tachyarrhythmia occurred in 10 patients (13.3%) after their final ablation.

**Figure 2:**
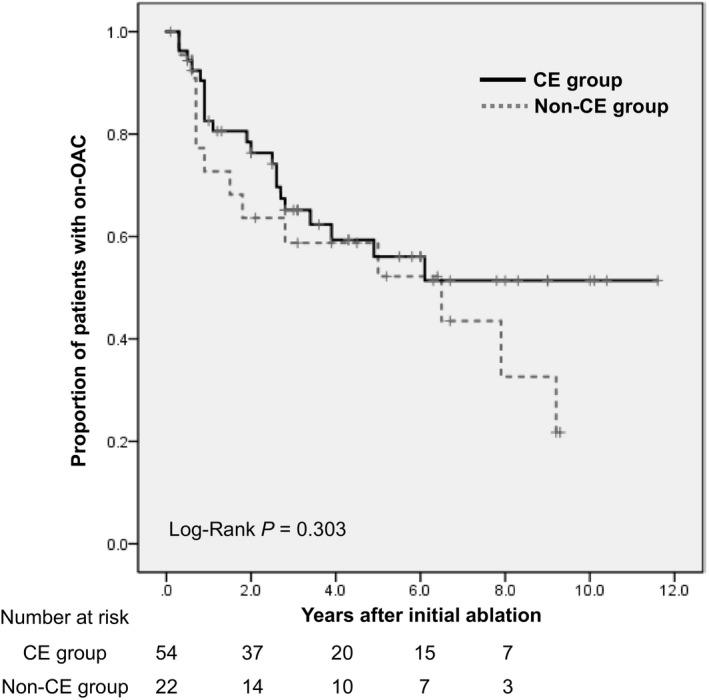
Persistence in use of oral anticoagulation therapy after initial catheter ablation according to stroke subtype. The proportion of patients receiving oral anticoagulation therapy by year is shown. CE, cardioembolic

**Figure 3:**
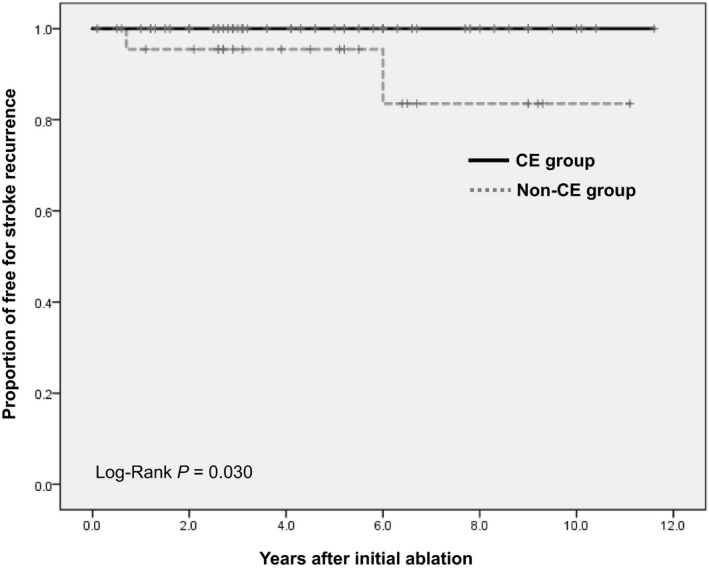
Kaplan‐Meier curve for recurrent ischemic stroke after catheter ablation according to stroke subtype. The proportion of patients free from stroke recurrence by year is shown. CE, cardioembolic

Oral anticoagulation was discontinued an average of 28.1 months following the initial ablation in 34 (44.2%) patients (21 [38.2%] and 13 [59.1%] in the CE and non‐CE groups, respectively; *P* = .129). These 34 patients had a median follow‐up of 1.8 (IQR 0.7‐3.6) years after discontinuation of OACs. Patients continuing OAC therapy in the CE and the non‐CE groups were 82.6% and 72.7%, 76.3% and 63.6%, and 56.1% and 52.2% at 1, 2, and 5 years, respectively (Figure 2). The continuation rate of OAC therapy stratified by the CHA_2_DS_2_‐VASc score is shown in Table 3. Given the concern for bleeding risks, the continuation rate of OAC therapy increased from the CHA_2_DS_2_‐VASc score 2 points to 5 points, but conversely it decreased at >6 points. The reasons for OAC discontinuation were prior non‐CE stroke in 8 patients, bleeding event/concern in 7, rhythm monitoring by cardiac implantable electronic devices (CIEDs) in 3 and by portable electrocardiogram in 2, and patient preference in 14 (Table 4). OACs were discontinued earlier in patients with bleeding events or those at risk for bleeding events than in other patients. The Kaplan‐Meier curves for recurrent stroke in the CE and non‐CE groups are shown in Figure 3. Importantly, none of the patients experienced recurrence of CE stroke in the CE group. In the non‐CE group, 2 patients exhibited recurrence of CVEs (log‐rank; *P* = .03); however, atrial tachyarrhythmia were not documented at the onset of the recurrent ischemic stroke (lacunar infarction in 1 and atherosclerotic stroke in 1 patient). One patient discontinued OAC at the time of recurrence, and another patient was receiving OAC. Case 1 was receiving aspirin, but Case 2 was not receiving aspirin. Kaplan‐Meier curves for recurrent stroke in on‐OAC vs off‐OAC could not be drawn because there was only 1 event in each group (*P* = .754).

**Table 3:** Continuation rate of oral anticoagulation therapy stratified by CHA_2_DS_2_‐VASc score

CHA_2_DS_2_‐VASc score							
On‐OAC/total (%)	2	3	4	5	6	7	Total
	1/5 (20.0%)	9/16 (56.3%)	15/25 (60.0%)	9/13 (69.2%)	7/13 (53.8%)	2/5 (40.0%)	43/77 (55.8%)

Abbreviation: OAC, oral anticoagulant.

**Table 4:** Reasons for discontinuation of oral anticoagulation therapy and outcome

Reasons for OAC discontinuation	n	Time of OAC discontinuation after final ablation (mo)	Use of antiplatelet drugs	ATa recurrence after final ablation	Recurrent stroke	Time of stroke after OAC discontinuation
Prior non‐CE stroke	8	27.2 ± 31.8 median 13	2 (25%)	None	1 (lacunar)	12 mo
Bleeding	7	24.2 ± 26.7 median 10	5 (71%)	None	None	
CIED monitoring	3	44.0 ± 55.8 median 13	3 (100%)	None	None	
Portable ECG monitoring	2	31.6 ± 1.3 median 32	0	None	None	
Patient preference	14	26.7 ± 20.2 median 27	2 (14%)	1 (CHA_2_DS_2_‐VAS_C_ = 2)	None	

Abbreviations: CE, cardioembolic; CIED, cardiac implantable electronic device; ECG: electrocardiogram; HR: hazard ratio; OAC, oral anticoagulant; PV: pulmonary vein.

### 3.6 Cases

We describe 2 cases of recurring ischemic stroke after successful AF ablation.

#### 3.6.1 Case 1

A 52‐year‐old woman with symptomatic drug‐refractory paroxysmal AF and prior lacunar infarction was referred to our hospital for catheter ablation. Antithrombotic therapy with warfarin and aspirin was continued after initial catheter ablation. A second ablation was performed for AF recurrence, with no subsequent events over the 4‐year follow‐up. The patient discontinued OACs and continued only aspirin; however, 6 years after the first ablation, a new cerebral infarction recurred. The subtype of recurrent stroke was lacunar infarct, and antiplatelet therapy was changed from aspirin to clopidogrel and no recurrence was observed for 3 years.

#### 3.6.2 Case 2

A 72‐year‐old man with symptomatic paroxysmal AF was referred to our hospital for catheter ablation. Embolism risk factors were absent except for age and preventive therapy with AAD was started. Although the palpitation attack disappeared after administration of drugs, a transient dysarthria appeared after 3 months. Before transfer to the stroke center by ambulance, the speech disorder improved and the MRI study revealed a small acute infarction due to atherosclerosis. Prior to this stroke, the patient had been completely unaware of palpitations and the electrocardiogram showed sinus rhythm. OAC therapy was introduced, and catheter ablation for AF was performed. The patient followed an uneventful course without complications and anticoagulation therapy with dabigatran was continued. Ten months later, speech disturbance and cerebral infarction recurred. Again, there was no evidence of AF, the cause of cerebral infarction was diagnosed as hemodynamically significant cerebral artery narrowing, and antiplatelet therapy was added.

### 3.7 Antiplatelet therapy

Antiplatelet agents (aspirin in 12, clopidogrel in 8 including a dual antiplatelet in 2; combination with OACs in 6) were initiated due to coexistence of cardiovascular diseases, prior lacunar infarction, or unknown reasons in 12, 3, and 3 patients, respectively. No significant differences in antiplatelet therapy were found between 2 groups (CE group, 12/55 [21.8%] vs non‐CE group, 6/22 [27.3%]; *P* = .766).

### 3.8 Bleeding events and anticoagulation management

Overall, 11 patients (14.3%) experienced bleeding events during follow‐up (7 gastrointestinal bleeding, 3 epistaxis, 1 chronic subdural hematoma, 2 anemia of unknown cause; incidence: 3.1/100 patient‐years). No significant difference was noted in HAS‐BLED scores between patients with a bleeding event and those without (2.3 ± 0.8 vs 2.0 ± 0.7; *P* = .196). Of the 53 patients taking warfarin as initial treatment, 14 continued on the same medication at the final observation and 17 switched to DOACs (due to bleeding events in 3 patients, triple antithrombotic therapy for coronary artery disease in 1, patient preference or doctors’ recommendation in 13). The average CHA_2_DS_2_‐VASc was 4.47 ± 1.33 for patients switching to DOACs (n = 17), 4.36 ± 0.93 for patients who remained on warfarin (n = 14), and 3.77 ± 1.44 for patients who stopped anticoagulant therapy (n = 22). Although not statistically significant, higher risk of embolism appeared to be a reason for switching drugs. Furthermore, lower risk may have led to termination of OAC therapy.

## 4 DISCUSSION

This study showed (a) a low incidence (0.54/100 patient‐years) of cerebral infarction recurrence in patients with a history of ischemic stroke who underwent AF ablation; (b) CE was the cause of approximately 70% of prior cerebral infarction, many patients had no symptoms of AF, and the LA was enlarged compared to non‐CE patients; and (c) a non‐CE mechanism (lacunar infarction and atherosclerotic stroke) was the cause of stroke in 2 patients with recurrent CVEs without atrial tachyarrhythmia recurrence.

### 4.1 Proportion of prior stroke patients in ablated patients and procedure safety

In reports from National Registry data of catheter ablation for AF, 5%‐10% of patients had a history of ischemic CVEs, with 8.1% reported in Japan,^8^ 4.5% in Europe,^9^ and 8.7% in the United States.^10^ In observational studies with a small number of patients,^11^, ^12^ a history of stroke did not predispose the patients undergoing AF catheter ablation to a significant risk of periprocedural CVE recurrence. In this study, the proportion of prior stroke patients was 9.3% among patients who had undergone catheter ablation for AF, and the rates of periprocedural complications were acceptable with no thromboembolic events observed.

### 4.2 Risk reduction of stroke after ablation and discontinuing OAC therapy

Previous observational studies have suggested an association between catheter ablation and a reduction in stroke risk.^2^, ^3^, ^4^ Furthermore, several recent reports have indicated that patients discontinuing OACs after successful ablation may experience a low stroke rate.^13^, ^14^, ^15^ This finding is supported by a comprehensive meta‐analysis that clarified the efficacy and safety of OAC discontinuation after successful AF catheter ablation.^16^ This meta‐analysis of 25 177 patients who underwent catheter ablation from 16 observational studies showed no difference in the occurrence of CVE between patients not taking OAC and those taking OAC after AF ablation (117 [0.9%] in the group without OAC and 135 [1.1%] in group with OAC therapy). Furthermore, the pooled analysis showed no statistically significant difference in the risk of CVE between patients on OAC and those off OAC, whether their CHADS2 was <2 or ≥2.

The proportion and timing of discontinuation of OAC therapy varies according to different reports and is important when considering stroke prevention strategies. In this study, OACs were discontinued in 44.2% of patients, and the decision to discontinue OACs was based on the patient's condition after confirming that arrhythmia was not recorded using a general monitoring method (24 hour recording every 3‐6 months) in an average 2‐year follow‐up. Although it is unclear whether the 2 year period was sufficient, we believe that these observations were more thorough compared to previous reports. More aggressive anticoagulation therapy interruption (eg, 3 months after ablation) was not attempted because we assumed that personalized antithrombotic therapy depending on the etiology of the ischemic stroke would lead to better outcomes.

### 4.3 Recurrence stroke after ablation in patients with prior stroke

Patients presenting AF and who have experienced a stroke are at a higher risk of developing recurrent stroke. Among patients with AF and a prior CVE, patients undergoing ablation have lower rates of recurrent stroke than AF patients not ablated. Bunch et al reported the 5 year impact of catheter ablation for AF in patients with a prior stroke.^17^ The 5 year risk of CVE (HR = 2.26; *P* < .0001) was higher in non‐ablated AF patients than in ablated AF patients. In this study, a low incidence (0.54/100 patient‐years) of ischemic stroke recurrence occurred in patients with a prior stroke following AF ablation, indicating the favorable impact of catheter ablation as secondary prevention. Despite advancements in medical therapy, the risk of recurrent ischemic stroke treated with optimal medication therapy remains between 6.5% and 20% per year in Asia.^18^, ^19^, ^20^, ^21^ The incidence rate per 100 person‐years of recurrent stroke in our study (0.543) was lower than that reported by Ogata and Omori (6.486 and 10.672, respectively).

### 4.4 Subtype of recurrent ischemic stroke and antithrombotic therapy

Hata et al showed higher recurrence rates of stroke in a Japanese community than in western populations.^22^ The recurrence rates at 1, 5, and 10 years were 7.2%, 30.4%, and 46.8% after lacunar infarction; 14.8%, 42.0%, and 46.9% after atherothrombotic brain infarction; and 19.6%, 42.2%, and 75.2% after CE stroke, respectively. While the role of OACs in CE stroke has been established, it may not prevent recurrence in other stroke subtypes, even in the absence of recurrent AF. In our cohort, the most common stroke etiology was CE (71.4%); however, approximately 30% of patients presented other causes for which antiplatelet therapy may have been indicated to prevent stroke recurrence. Evans et al reported the recurrence rate of stroke in 386 acute stroke patients with AF treated with warfarin or aspirin (the latter used in patients with contraindications or if anticoagulation therapy was refused) for each etiology.^23^ The increased stroke rate with aspirin was due predominantly to CE recurrence in patients initially presenting CE stroke (8.4% vs 1.9%; *P* = .01), while the recurrence rate in aspirin‐treated patients presenting lacunar infarction and AF was similar to patients receiving warfarin (8.8% vs 8.9%). Mechanisms of ischemic stroke and the effects of antithrombotic treatment have also been reported.^24^ Of 217 ischemic strokes occurring in 3950 participants in the Stroke Prevention in AF I‐III clinical trials, 52% were classified as likely CE, 24% as non‐CE, and 24% of uncertain cause (ie, 68% of classifiable infarcts were deemed CE). Compared to those receiving placebo or no antithrombotic therapy, the proportion of CE stroke was lower in patients taking warfarin (*P* = .02), while the proportion of non‐CE stroke was lower in those taking aspirin (*P* = .06). Most (56%) ischemic strokes occurring in AF patients taking adjusted‐dose warfarin were non‐CE; thus, aspirin in AF patients appeared to primarily reduce non‐CE strokes.

### 4.5 Study limitations

This study was a small sample, single‐center, non‐randomized retrospective study where interruption or continuation of OAC therapy was left to the discretion of the physician. Attempting to distinguish which stroke subtypes would respond to a particular strategy is a great problem for small retrospective sample sizes; therefore, a large prospective study is warranted. Since recurrence of cerebral infarction was not routinely screened using imaging findings such as MRI, cerebral infarction with asymptomatic, or poor symptoms may have been overlooked. An intracranial and/or extracranial arteriography by MRI or ultrasonography was not routinely performed for all patients to assess atherosclerotic involvement. Asymptomatic AF recurrence is a very important issue for patients with a high risk of embolism. Even in patients presenting with highly symptomatic AF, asymptomatic episodes may occur and significantly increase after ablation. Conventional follow‐up such as 24‐hour Holter monitoring is not sufficient to identify asymptomatic AF recurrences after ablation. In this retrospective study, assessment for AF recurrence was performed with rhythm monitoring by CIEDs in 3 patients and well‐trained self‐monitoring using portable electrocardiogram in 2 patients. We believe that this problem may be solved by advances in wearable monitoring technology.

## 5 CONCLUSIONS

In patients who underwent catheter ablation for AF, the incidence of recurrent stroke was 0.54/100 patient‐years for individuals with prior stroke. While prior stroke in some cases may not have been due to AF, careful follow‐up with optimal antithrombotic therapy is needed to minimize the risk of recurrent stroke following catheter ablation.

## CONFLICT OF INTEREST

Authors declare no conflict of interests for this article.
